# Advances in the Clinical Diagnostics to Equine Back Pain: A Review of Imaging and Functional Modalities

**DOI:** 10.3390/ani14050698

**Published:** 2024-02-23

**Authors:** Natalia Domańska-Kruppa, Małgorzata Wierzbicka, Elżbieta Stefanik

**Affiliations:** Department of Large Animal Diseases and Clinic, Institute of Veterinary Medicine, Warsaw University of Life Sciences, 02-787 Warsaw, Poland; natalia_domanska-kruppa@sggw.edu.pl (N.D.-K.); malgorzata_wierzbicka@sggw.edu.pl (M.W.)

**Keywords:** back pain, chronic pain, horse, EMG, FES

## Abstract

**Simple Summary:**

Back pain in horses is a common occurrence and has been considered to be an important factor affecting the well-being of horses. Its occurrence may be related to soft-tissue lesions, osseous injuries, tack-associated problems, or neurological disorders. In addition to conventional diagnostic methods, there are also more sophisticated ones, such as scintigraphy or computed tomography. Advanced diagnostic modalities include algometers, determination of lameness with inertial sensor systems, biometric mats, and geometric morphometrics methods, electromyography, as well as functional electrical stimulation. The purpose of this review article is to summarize the main back diseases in horses, as well as to provide an overview of their traditional, but also modern diagnostic methods. Although traditional diagnostic methods remain the foundation of orthopedic examination and back diseases, the use of new methods will allow for a better understanding of the pathogenesis of these diseases and will also allow for the objective monitoring of the rehabilitation and training process.

**Abstract:**

Back pain is common in ridden horses. Back diseases in horses include Impinging Dorsal Spinous Processes, Ventral Spondylosis, Osteoarthritis of Articular Process, Intervertebral Discs Disease, Vertebral Fractures, Conformational Abnormalities, Desmopathy of the Supraspinous Ligament, Desmopathy of the Intraspinous Ligament, and Longissimus Muscle Strain. Back pain may also develop as a result of lameness (particularly hindlimb lameness). A poorly fitting saddle and an unbalanced rider are also considered important factors influencing the development of back pain in horses. The conventional diagnosis of equine back pain includes a clinical examination and diagnostic imaging examination using ultrasound, radiography, and thermography. Advanced diagnostic modalities of equine back pain involve the objectification of standard procedures through the use of algometers, a lameness locator, biometric mats, and the geometric morphometrics method. In addition to modern diagnostic methods, such as computed tomography and scintigraphy, advances in the diagnosis of equine back pain include the use of electromyography and functional electrical stimulation. The aim of this review article is to familiarize clinicians with the usefulness and capabilities of conventional diagnostic protocols and advanced diagnostic modalities. Although orthopedic examination and traditional diagnostic methods will remain the foundation of the diagnosis of back diseases, modern methods meet the growing expectations towards high-performance horses and allow for deeper diagnostics and objective monitoring of rehabilitation and training progress.

## 1. Introduction

The equine industry has been continuously and intensively developing over the last few decades, presenting researchers and clinicians with increasing challenges to keep up with the demands of high-level horse sports as well as widespread leisure riding. The sport’s increased popularity and widespread presence on social media have led to frequent criticism from audiences due to concerns about equine welfare. Modern conceptualizations of welfare issues associated with equestrian sports and leisure riding emphasize the great need for the early and effective recognition of any gait abnormalities or lameness to prevent injuries and chronic discomfort when ridden [1,2]. It is important to note that tack or rider-induced back pain, one of the significant causes of back diseases, is closely related to overloading the back during riding. Similar to how being overweight is considered to be a disease in human civilization, in the case of mainly leisure horse riding, it also becomes a contributing factor to diseases in equine civilization [3].

More than 47% of sport horses at normal work suffer from unrecognized lameness related to back pain [4]. Between 48% and 54% of horses in dressage, showjumping, and eventing show signs of back disease [5]. Similarly, 55% to 74% of leisure and riding school horses are severely affected by back disorders and riding school horses are twice as often affected with back pain than other working horses [6]. Finally, 85% of national hunt racehorses and 90% of flat racehorses exhibit clinical signs of back pain [5]. Therefore, back pain is considered one of the most common syndromes in ridden horses, responsible for chronic pain, poor performance, behavioral issues, impaired ability to work, or nonspecific lameness [7,8]. Experiencing chronic pain may cause changes in the horse’s behavior and may also result in the development of additional diseases such as gastric ulcers [9,10]. The reduced accessibility of the affected back area, the need for using sophisticated clinical protocols and diagnostic modalities, and the necessary comprehensive experience of the clinician make an accurate identification of the primary pathology challenging [8,11].

Therefore, given the significant impact of back pain on horse performance, the use of advanced diagnostic modalities and functional therapies has become the desired direction of development in equine medicine. This review aims to familiarize clinicians with the usefulness and capabilities of conventional diagnostic protocols and advanced diagnostic modalities for the equine back region, aiming to enhance their current clinical applications in equine medicine.

## 2. The Main Cases of Equine Back Pain

Back diseases in horses are mainly associated with soft-tissue lesions, osseous injuries, tack-associated problems, or neurological disorders [12]. Primary cases include Impinging Dorsal Spinous Processes (IDSP, ‘kissing spines’), Ventral Spondylosis (VS), Osteoarthritis of Articular Process (OAAP), Intervertebral Discs Disease (IVDD), Vertebral Fractures (VF), Conformational Abnormalities (CA), Desmopathy of the Supraspinous Ligament (DSSL) and/or Desmopathy of the Intraspinous Ligament (DISL), and Longissimus Muscle Strain (LMS). Moreover, lameness, particularly hindlimb lameness, can cause secondary back pain [4,13]. Finally, the rider or tack can also exacerbate problems occurring in the back region through poor saddle fit or riding skills, unsuitable training techniques, lack of fitness/strength of the horse, or excessive rider weight [3,14]. Therefore, in the evaluation of equine back pain, all these cases should be considered.

### 2.1. Impinging Dorsal Spinous Processes (IDSP, ‘Kissing Spines’)

IDSP occur when vertebrae in the spine are too close together, rather than being spaced apart as in a healthy spine. The exact pathogenesis has not been clearly defined to date; it can also be secondary to other back pain issues. IDSP have mostly been observed in Thoroughbreds and performance horses with jumping and dressage horses having the highest incidence of lesions [15]. Dorsal spinous process impingement is radiographically characterized by a close approximation of adjacent spinous processes with reactive bone sclerosis affecting these spinous processes [5,16]. In more advanced cases, severe overriding can result in a periosteal reaction, pseudodarthrosis, misshapen dorsal summits, and even the fusion of adjacent dorsal spinous processes can occur [5,17]. More severe changes may be accompanied by soft tissue injuries (see below: desmopathy of the supraspinous and intraspinous ligament). Among clinical signs, the most common are resistance to grooming, saddling and girthing, pain on palpation of thoracolumbar region, vague lameness, hypomobility of the spine and overall poor performance [18,19].

### 2.2. Ventral Spondylosis (VS)

VS occurs when new bone formation caused by an ankylosing or degenerative disease of the ventral joints appears on the ventral aspect of the vertebral bodies in the thoracolumbar region of the spine. It can be apparent as an osseus bridge between adjacent vertebral bodies or as bony growths that impinge on each other [20,21]. Pathogenesis is thought to be connected with other back diseases such as chronic ventral longitudinal ligament degeneration [22]. The clinical signs of VS are poorly documented to date, ranging from acute pain or chronic back stiffness [23]. However, in some cases clinical signs may be more serious when the osteophytes compress nerve roots at the intervertebral foramen or grow into the vertebral canal and compress the spinal cord [24,25].

### 2.3. Osteoarthritis of Articular Process (OAAP)

OAAP occurs when the presence of additional osseous abnormalities appears on the intervertebral joints. Among the disciplines, show jumpers were less affected than other horses [26]. The main clinical signs when assessing at stance and palpating are thoracolumbar muscle soreness and atrophy, tension, spasm, and reluctance to lateral flexion. During movement evaluations, OAAP presents as overall full body stiffness, reduced hindlimb impulsion, and toe drag [27]. In horses with concurrent IDSP, additional clinical signs may include epaxial muscle atrophy, problems with picking up, or hyperbolic lifting of the hindlimbs [26].

### 2.4. Intervertebral Discs Disease (IVDD)

IVDD refers to the two main diseases considered to be inflammatory (discospondylitis) and degenerative (discospondylosis) conditions. Discospondylitis is thought to be rare in horses; however, the introduction of modern diagnostic imaging techniques and increased awareness of its occurrence in horses have changed this point of view [28,29]. Discospondylosis appears more often being related to the increased dorsoventral mobility and the increased disc thickness of these joints [30]. Clinical signs related to IVDD are reported in severe cases in the form of reduction in spine movement, ataxia of forelimb, or lameness [31]. A dramatic epaxial and gluteal muscle atrophy associated with thoracic IVDD was also noted [28].

### 2.5. Vertebral Fractures (VF)

VF may occur within dorsal spinous processes of the withers or vertebral bodies/articular surfaces anywhere along thoracolumbar spine. A pre-existing pathology of the vertebral junction and mild repetitive overuse injuries may be predisposing factors for fatal lumbar vertebral fractures. Catastrophic falls during jumping, high-speed falls, steeplechase races, or polo collisions may lead to vertebral bodies fractures [32], and could have terrible consequences such as immediate and variable degrees of paraparesis or paraplegia if the spinal cord receives damage [15]. Fortunately, in most cases, the injury is not that extensive and the spinal cord remains unaffected. Then local pain and swelling are usually observed and the prognosis is good without a need for surgical treatment [33,34]. Clinical signs depend on the degree of neurological compromise and if the spinal cord is not affected severe back pain may be the only clinical manifestation. Minor local neurological signs can lead to epiaxial muscle wastage and severe spinal cord damage leads to paralysis or recumbency [15].

### 2.6. Conformational Abnormalities (CA)

Conformational abnormalities include mainly scoliosis, kyphosis, and lordosis. Scoliosis is an uncommon lateral deviation of the vertebral column attributed mostly to congenital vertebral malformations [35]. Among the clinical signs, characteristic posture, stiff hindlimb gait, and inflexibility of the back were described [15]. Kyphosis is a dorsal deviation of the thoracolumbar spine and has been reported in combination with scoliosis. Acquired kyphosis may be a result of traumatic injury, compression fractures of vertebral bodies, or chronic bilateral hindlimb pain [36]. Lordosis is a ventral curvature of the spine in the mid- or caudal-thoracic region externally visible as a severe downwards curve of the topline. As the epaxial structures alongside the vertebral column undergo extra stress at the lordosis site these horses are predisposed to back pain. They are presented with signs of overall expressed poor performance due to soft tissue damage at the back when ridden. This condition may have varying degrees of advancement and be congenital or develop as a degenerative trait associated with ageing [15].

### 2.7. Desmopathy of the Supraspinous Ligament (DSSL) and/or Desmopathy of the Intraspinous Ligament (DISL)

DSSL and DISL refer to injuries of the supraspinous ligament (SSL) and/or interspinous ligament (ISL). The SSL is the extension of the nuchal ligament in the thoracolumbar region which forms attachments on each summit of the dorsal spinous processes along the thoracolumbar spine and receives multiple tendon insertions of longissimus dorsi that support its structure [37]. The ISL is composed of four ligamentous sagittal layers that are also adjacent to the spinous processes and blend into the SSL dorsally [38]. Both ligaments have a stabilizing function for the thoracolumbar spine due to a close connection between the SSL, ISL, and the myofascia of the epaxial muscles. High level sport horses are more prone to injuries of the SSL with most reports involving racehorses and showjumpers [39]. Potential causes of DSSL/DISL include strains during ventroflexion, compressive injuries due to saddle pressure, enthesiopathies at the insertion sites, avulsion fractures, or secondary pathologies due to underlying conditions, for example, overriding dorsal spinous processes [40].

### 2.8. Muscle Related Back Pain

Damage to the soft tissues may be a common cause of back soreness in horses and may involve groups of the epaxial and hypaxial musculature [34].

Longissimus Muscle Strain (LMS) refers to damage or the breakdown of muscle fibers and occurs most often in large muscles of the hindlimbs or topline. LMS can result from primary back pathology, poorly fitting tack, an unbalanced rider, lameness elsewhere in the horse, bracing secondary to ulcerative gastric syndrome, poor fitness of core musculature, inappropriate training or inadequate warm up for the expected workload, or trauma [41]. Any strain within the m. longissimus dorsi, the largest muscle of the horse’s back, may lead to back pain as it is also in humans [42]. Clinical signs may concern poor performance, localized heat, swelling, and pain reaction by palpation. The horse’s behavior may be altered and thoracolumbar flexion or lateral flexion might be markedly reduced [43].

The multifidus muscle is one of the most important postural muscles in horses. Its caudal insertions occur on the lumbar mammillary processes, thoracic transverse processes, and cervical articular processes until reaching the lamina with short fasciles or the spinous processes with long fascicles [44]. Due to its direct connection with the joint capsule of the articular processes in the cervical region, it influences neuromotor control, proprioception, and joint stability of these joints [45]. Atrophy and loss of functionality of this muscle develops secondary to other pathological changes in the spine or dysfunction of the epaxial muscles in horses [46]. Moreover, it has been proven that the muscle remains atrophic after the initiating pain stimulus ceases [46,47]. As a result, the loss of functionality perpetuates the instability of the intervertebral joints and deepens the existing pain. Therefore, it is very important to implement rehabilitation and mobilization exercises aimed at reactivating and strengthening this muscle as a permanent element of the therapy for other back diseases [46,48].

The iliopsoas muscle is a deep hypaxial muscle, which consists of three muscles—psoas major, psoas minor, and iliacus muscle and its main role is to stabilize the spine in the lumbosacral area. The effects of its contraction are hindlimb protraction and hip joint flexion. It is considered to be the most important muscle involved in the engagement of the hindquarters [49]. The Iliopsoas muscle is responsible for moving the limb forward during the non-weight-bearing phase of gait. Due to its functions, strain of this muscle causes a lack of propulsion, weakening of adduction, limitation of lateroflexion movement of the spine in the sacrolumbar area, inability to step under, and resistance to collection [49,50]. Due to its location, is not accessible for palpation, and its dysfunction is manifested by bilateral sacroiliac and back pain. Iliopsoas strain is often underdiagnosed due to diagnostic difficulties and a low level of awareness among veterinarians.

### 2.9. Tack or Rider Induced Back Pain (TIBP/RIBP)

TIBP/RIBP may be a severe problem occurring in the back region. One of the most common reasons for TIBP is an ill-fitting saddle which can cause muscle soreness, abnormal behavior during grooming or tacking up, muscle atrophy, generalized back stiffness, shortening of the forelimb step length, or unwillingness to longitudinal bend [51]. Ill-fitting saddles are also associated with equine thoracolumbar region asymmetries which can exacerbate riders’ imbalance and back pain [4]. In RIBP cases, weight distributed on the horse’s back can induce an overall extension of the back what may contribute to soft tissue lesions [52]. The adverse effects of the change in back loading were apparent with overweight riders especially if the tack was not ideally fitted [3]. Force distribution on the horse’s back may also be influenced by the rider’s core strength, limb muscle strength, as well as coordination and balance [53,54,55]; however, this aspect of TIBP/RIBP may be objectively evaluated using a biometric mat. One may observe that riders take care about their own fitness level as this has a big impact on the quality of their riding. A lack of balance and core strength is likely to be detrimental to their horse’s performance [56]. A fatigued or unbalanced rider causes an increased and uneven load on the horse during ridden work which will increase the physiological demand of exercise [57], as well as an overload of the bone elements of the vertebral column, ligaments, and muscles, which may lead to severe spine diseases [3,51].

## 3. Conventional Diagnostic Protocol of Equine Back Pain

Conventional diagnosis of equine back pain includes a clinical examination and diagnostic imaging examination using ultrasound, radiography, and thermography [8,11]. Reports on the use of the conventional diagnostic protocol in the equine back pain evaluation are summarized in Table 1.

There are several published protocols for the clinical examination of horses for back problems [7,58,59,60]. Acquiring a good comprehensive history is crucial in all cases of back pain, as clinical symptoms are varied, multiple, and easily confusable with a number of other clinical problems. The next step is a complex clinical examination at rest, which needs to be carried out carefully, including the examination of the whole horse for other causes of lameness and loss of performance problems. The inspection at rest includes observation and back palpation [61]. The initial inspection follows with the horse standing ‘four square’ on a flat hard surface to carry out a general appraisal of the conformation of its back, fore and hind limbs, as well as assessing its general condition and muscling. The assessment of the symmetry of the pelvis and hindquarter is extremely important in differentiating a pelvic or hindlimb problem. One of the most important parts of the clinical examination is palpation of the thoracolumbar spine. Spasm or guarding of any of the musculature of the thorax and lumbar regions may be part of a primary problem or an indicator of deeper pain [62]. The next step is a clinical examination in exercise. The horse should be watched in walk and trot in hand on a straight line, then on small and large circles on hard ground. Following the in-hand examination, the horse should be lunged in three gaits in both directions to aid in ruling out lameness. Subsequently, the saddle should be checked for a proper fit to the individual horse. Then, a clinical examination in walk and trot should be repeated under saddle. Observing the horse under saddle or in harness should also be part of a work-up for thoracolumbar problems, and stiffness or reluctance to bend in either direction can be reported by the rider. Flexion tests may also be performed. Horses with suspected back pain should always undergo a basic neurological examination to exclude a primary neurological problem as a cause of back pain or altered gait/lameness [63].

Ultrasonography (US) is most helpful in evaluating the SSL, ISL, muscles of the top line, as well as the spinous processes and facet joints of the thoracolumbar spine. With transducer frequencies between 2.5 and 7.5 MHz, most pathologies affecting soft tissues can be successfully diagnosed. Performing ultrasound in this region requires reliable knowledge about the anatomy of the muscles, tendons, ligaments, and joint surfaces [64]. When assessing in a strict median longitudinal and transverse plane, a great variability in the normal echogenic appearance of imaged structures should be considered. As the ligaments are elastic, hypoechoic areas may occur if the horse holds its head up, leading to the relaxation of the ligaments [65]. When examining the spinous processes, it should be kept in mind that the apices of Th3–Th7 vertebrae have separate centers of ossification and thus a roughened appearance. The remaining spinous processes have smooth surfaces. The interspinous space may be assessed between two adjacent spinous processes depending on their angulation. Placing a convex probe approximately 2–4 cm paramedian enables the evaluation of the left and right surfaces of the spinous processes for the presence of new bone formations or fractures [66]. The facet joints of the thoracolumbar spine are also evaluated in a transverse and longitudinal plane. The easiest way to identify the site of probe placement is the last rib, as it is easy to palpate and corresponds to the facet joint of Th17–Th18 vertebrae. Cranial and mid-thoracic facet joints are more difficult to identify as they are located much deeper. Once the facet joint of Th17–Th18 vertebrae has been localized, adjacent joints cranially and caudally are examined for left–right symmetry, width, and new bone formations [67,68].

Radiography (X-ray) is one of the fundamental diagnostic methods for assessing bony structures suspected of pathological changes. Most portable X-ray units can produce high-quality radiographs. Radiographs should be taken under sedation to reduce motion, and the horse should stand squarely on all four legs to avoid spine rotation. The use of radio-opaque markers helps correlate radiographic changes with specific locations on the horse. Key findings related to pathological changes in the thoracolumbar spine include impinging dorsal spinous processes, sclerosis, osteolysis, intra and periarticular remodeling, and vertebral fractures. Since the appearance of radiographic symptoms rarely corresponds with the degree of clinical signs, other functional diagnostic methods are essential to improve the accuracy of the diagnosis [69].

Infrared thermography (IRT) is an additional diagnostic method that detects the radiated power emitted from the body surface and provides data about surface temperature [70]. IRT imaging is performed non-contact using a thermal imaging camera. It is crucial to remove sand and dirt from the horse’s back and rump before imaging, preferably no later than 30 min before imaging, as the heat released during cleaning may affect the obtained image. The distance from which the horse is imaged, the emissivity of the thermal camera, and the method of image processing are also important considerations [71]. Basic image evaluation includes point, linear, or area measurement of minimum, maximum, and/or average temperature in the region of interest (ROI) corresponding to selected areas of the back [72,73,74]. However, modern analytical approaches, such as image texture analysis [75,76,77], image entropy analysis [78], and the application of a Pixel-Counting Protocol [79,80] are also available. The main disadvantage of IRT imaging is its low specificity due to the influence of factors such as the thickness of the skin and subcutaneous tissue [81], the length of the hair, external temperature [73,82], humidity of both the air and the horse’s body, and sunlight at the imaging location [71]. Since IRT is sensitive to various internal and external factors, it is commonly used for assessing the horse’s welfare [83], physiological response to effort [77], saddle fit [72,73], and the impact of rider–horse interaction, including matching [74] and the effect of bodyweight [18,76,78,84], rather than diagnosing back diseases.

**Table 1:** The selected diseases of the equine back area and their main symptoms and signs which were identified using the conventional diagnostic protocol.

Disease	Examination	Symptoms/Signs	Refs.
IDSP	Clinical	Resistance to grooming, saddling and girthing, pain on palpation of thoracolumbar region, poor performance	[18,84]
	US	Narrowing of the interspinous space, irregularity in bone surface of the spinous processes	[66]
	X-ray	Narrowing of the interspinous space, increased opacity of the margins and/or radiolucencies and/or osteolysis of the spinous processes, impinging/overlapping/fussion of the spinous processes, modeling of the dorsal or cranial aspect of the spinous processes, change in shape of the spinous processes	[85]
	IRT	“hot streak” perpendicular to the thoracic spine, “cold streak” perpendicular to the thoracic spine, combination “hot spot”–“cold streak” pattern over the back	[86]
VS	Clinical	Acute pain or chronic back stiffness, neurologic deficits, lameness, signs of pain after excercise	[23,24]
	US	Not applicable	-
	X-ray	Presence of the osteophytes, bridging intervertebral space (evidence of the increased opacity at opposing borders)	[23]
	IRT	Not applicable	
OAAP	Clinical	Thoracolumbar muscle soreness and atrophy, tension, spasm, reluctance to flex, stiffness, reduced hindlimb impulsion and toe drag	[27]
	US	Degree of periarticular modeling (reduced definition of the hyperechoic dorsal horizontal border of the APJ, irregularity of the dorsal border) and APJ enlargement (extension beyond the level of the mamillary body or the dorsolateral aspect of the articular process), joint space presence or narrowing	[87]
	X-ray	Periarticular new bone formation on the dorsal or ventral margins of the APJ, reduction of the interforaminal space	[88]
	IRT	Not applicable	
IVDD	Clinical	Reduction in spine movement, ataxia of forelimb or lameness, epaxial and gluteal muscle atrophy, muscle weakness, muscle twitching	[28,31,89,90]
	US	In cases of ventrolateral vertebral body spondylosis elevations of the margins of the vertebral bodies with nonvisualization of the disc space and intervertebral disc; irregular endplates or bone surfaces, widened or narrowed disc spaces, the ability to see into the depth of the disc space, vertebral step formation	[89]
	X-ray	Vertebral endplate osteolysis and sclerosis, vertebral subluxation, widening or collapsed of the intervertebral space, degenerative changes	[89,90]
	IRT	Not applicable	**-**
VF	Clinical	Local pain and swelling, epiaxial muscle wastage, severe spinal cord damage leads to paralysis or recumbency	[15,33,34]
	US	Localised disruption to the surface of the bone	[33]
	X-ray	Visualization of the fracture line, displacement of fracture fragments	[33]
	IRT	Not described	**-**
CA	Clinical	Stiff hindlimb gait and inflexibility of the back, poor performance	[15]
	US	Not described	**-**
	X-ray	Abnormal alignment of the vertebrae	[91]
	IRT	Not described	**-**
DSSL/DISL	Clinical	Back pain, localized thickening, sensitivity to palpation, gait restriction, muscle atrophy	[92]
	US	Irregularity of the spinous processes, anechoic focus in the ligament	[61,92]
	X-ray	Not applicable	**-**
	IRT	Hot spots along dorsal midline	[61]
LMS	Clinical	Poor performance, localized heat, swelling, pain reaction by palpation, altered behaviour, reduced thoracolumbar flexion or lateral flexion	[43]
	US	Focal hypoechoic areas	[92]
	X-ray	Not applicable	
	IRT	Hot spots along longissimus dorsi muscle or focal increased temperature	[61]
TIBP/RIBP	Clinical	Muscle soreness, abnormal behaviour, muscle atrophy, generalized back stiffness, shortening of the forelimb step length, unwillingness to bend	[51]
	US	Not applicable	**-**
	X-ray	Not applicable	**-**
	IRT	Possibility to visualize the influence of the rider on the horse and the saddle fit	[72,73,74]

CA, Conformational Abnormalities; Clinical, Clinical examination; DISL, Desmopathy of the Intraspinous Ligament; DSSL, Desmopathy of the Supraspinous Ligament; IDSP, Impinging Dorsal Spinous Processes; IRT, infrared thermography; IVDD, Intervertebral Discs Disease; LMS, Longissimus Muscle Strain; OAAP, Osteoarthritis of Articular Process; RIBP, Rider Induced Back Pain; TIBP, Tack Induced Back Pain; US, ultrasonography; VF, Vertebral Fractures; VS, Ventral Spondylosis; X-ray, radiography; Refs., References.

## 4. Advanced Diagnostic Modalities of Equine Back Pain

### 4.1. Objectification of the Conventional Diagnosis Protocol

Some parts of this routine protocol for the conventional diagnosis of equine back pain are assessed subjectively. Therefore, methods for objectifying the diagnosis are needed. During a comprehensive clinical examination at rest, an algometer may be employed to render the inherently subjective back palpation more objective. The algometer is a small, hand-held tool used to assess the level of pressure a patient can tolerate in a specific area, known as the mechanical nociceptive threshold or pressure pain threshold of that area. Algometry enables the objective measurement of musculoskeletal pain thresholds [93,94] with acceptable repeatability [95]. This tool aids in localizing the pain site and comparing the response to treatment. However, further research is needed to fully realize the potential value of the algometer as a clinical tool in the diagnosis of back diseases. During a comprehensive clinical examination in walk and trot in hand, the Lameness Locator (LL, currently known as Equinosis Q) may be used to objectively confirm the presence or absence of lameness. The LL is a system of wireless inertial sensors positioned on the head poll, the pelvis, and the right front pastern. This tool provides results for the fore- and hindlimbs, categorizing lameness from mild to moderate/severe and aiding in the recognition of type, degree, and location of lameness [96]. With increasing awareness that trotting in hand may not be sufficient for diagnosing lameness and that horses can exhibit lameness when ridden, the examination protocol should always include the evaluation of horses under tack [97,98,99]. Therefore, a comprehensive clinical examination in walk and trot under saddle may be improved using the Biometric Mat (BM). The biometric mat is a tool that allows for the capture of the pressure distribution on the horse’s back, assessing the complex interaction of forces between horse, saddle, and rider. As it can be used in a standing position, walk, and trot, the holistic effect of the rider’s core strength, limb muscle strength, coordination, and balance on the horse’s back may be assessed. Moreover, the biometric mat was used to determine the effect of the horse–rider interaction on the horse’s welfare state [100], which can also be successfully reflected through the dorsal line posture using the Geometric Morphometrics (GM) method [6]. The GM method is a completely non-invasive analytical tool that allows for the quantification and comparison of the shape of a horse’s dorsal line of the back based on regular visible light photos [80,101].

### 4.2. Advancements in the Diagnosis of Equine Back Pain

Modern diagnostic modalities such as nuclear scintigraphy or computed tomography are promising in diagnosing difficult cases; however, are expensive and not easily available to the general practitioner [102]. Therefore, reports on the use of the potentially more available advanced diagnostic modalities were summarized in Table 2.

Despite the wide range of diagnostic modalities, pinpointing the exact cause of thoracolumbar pain in horses remains challenging. In humans, it has been confirmed that individuals with back pain exhibit early myoelectrical manifestations of muscle fatigue, and electromyography (EMG) serves as a useful tool for objective low back pain diagnosis. Muscle fibers are stimulated to contract by action potentials triggering depolarization and repolarization within individual muscle fibers, creating electromagnetic fields that can be measured by EMG. There are two methods of EMG: the first, needle EMG, is invasive and involves needle electrodes inserted into the muscles of interest, while the second, surface EMG (sEMG), uses electrodes applied to the extracellular skin surface above the muscles of interest. In recent research, only one group opted for the needle EMG technique to investigate the neuromuscular background of changes causing back pain [103], while all others focused on sEMG. The sEMG method has been employed to study back muscle activity during walk [104] and trot [105,106] as well as during jumping [107] and induced flexing [108]. Despite this research, the use of sEMG is still in its infancy in veterinary motion analysis. However, the application of modern devices is crucial to deeply understand many important aspects of this functional diagnostic modality. In IDSP, sEMG may be used to evaluate back and abdominal muscle weakness and malfunctioning nerve conduction, as unwillingness to bend a sore back is associated with weakness in these muscles and malfunctioning nerve conduction [18]. In OA, susceptibility to the development of the disease is supposed to be related to the biomechanics of this region, which involves lateral bending, axial rotation, and dorsoventral mobility [30]. As the activity of thoracolumbar muscles (m. longissimus dorsi and m. multifidus) is responsible for the mobility of this back segment, examining their function using sEMG seems to be a proper research direction in sound horses, those with clinical signs of OA, and during therapeutic exercises [109]. The assessment of m. longissimus electromyographic activity using sEMG should be performed at the level of the Th12 vertebra, where its maximum amplitude is concentrated [54,108]. The high maximum activity of the m. longissimus dorsi at Th12 could contribute to the development of muscle pain at this site [104]. In IVDD, sEMG would be a useful tool to determine the presence of a peripheral neurogenic component to muscle atrophy, as muscle atrophy may be mistakenly presumed to be associated with disuse [28]. In DSSL/DISL, the functional connection of the SSL and ISL with m. longissimus dorsi suggests that sEMG studies on this muscle could be helpful for confirming the clinical importance of diagnostic imaging signs as well as for the follow-up of treatment and rehabilitation [40]. In some desmopathies, altered collagen fiber alignment and arrangement of the ligamentous layers occur; therefore, it would be of clinical importance to confirm if the back muscles that have a functional connection with the overlying ISL show an altered sEMG signal in affected horses [110]. However, all these hypotheses require confirmation in further studies.

Functional Electrical Stimulation (FES) is another advanced diagnostic modality that should be investigated for its clinical usefulness in equine back pain diagnosis. FES involves the painless electrical stimulation of a single muscle or a group of muscles to restore or improve their function. It has been used in humans to reverse muscle atrophy in patients after spinal cord injuries. FES has also been widely applied in the rehabilitation of various injuries in horses, as well as for overall performance enhancement [111]. In addition to its therapeutic use, FES can serve as a diagnostic tool over the thoracolumbar, lumbosacral, and cervical regions of the horse to detect dysfunction in muscles’ neuromuscular control response. Observing the muscles’ movement during FES stimulation allows for the assessment of their symmetry and range of motion. If an abnormal movement pattern, such as hypermobility, hypomobility, or postural sway, appears, the cause of this deviation is further examined. This approach helps localize the sites of dysfunction or pain, which can be thoroughly investigated using other diagnostic procedures. The stimulation of a painful area with FES causes repeatable and constant unwillingness to specific movements, serving as valuable diagnostic information [112].

Finally, both these modalities may be applied in supporting the functional exercises of the equine back and thus improve the prevention and treatment of equine back pain. It is crucial to include exercises for back stabilization during rehabilitation and training to prevent the recurrence of pain signs after back pain treatment. The dorsal line (topline) of the horse runs from the withers along the back down to the croup, with the m. longissimus dorsi being the main muscle unit. M. longissimus dorsi stabilize the spine, facilitate proper locomotion, and provide support to the saddle and rider. As the dorsal line is part of the active epaxial musculoskeletal system, its influence on horse performance is evident [113]. Some ground training with poles and the use of lunging aids, such as a chambon, rubber bands, side reins, and a Pessoa, have been recommended to improve back muscle action [74,80,109,114]. However, further research is needed to explore the increase in the ability of m. longissimus dorsi to stabilize the back after the use of lunging aids. Incorporating objective measurement modalities, as described above, into functional exercise monitoring will shed new light on the prevention and treatment of back pathology and the rehabilitation of horses with back pain.

**Table 2:** The selected diseases of the equine back area and their main signs which were identified using the advanced diagnostic modalities. When there are no recent signs, this summary was extended to justify the potential use of each advanced diagnostic modality.

Disease	Modality	Signs	Possible Use	References
IDSP	Algometry	-	To assess the intensity of back pain	[115]
	LL/BM/GM		To evaluate back pain and secondary lameness	[116]
	sEMG	To evaluate back and abdominal muscle weakness and malfunctioning nerve conduction	-	[18]
VS	Algometry	-	To assess the intensity of back pain	[115]
	LL/BM/GM	-	To evaluate back pain and secondary lameness	[116]
	sEMG	-	EMG is important in the differential diagnosis of cervical spondylosis—shows degrees of denervation and the number of roots involved, but has no prognostic value	[117]
OAAP	Algometry	-	To assess the intensity of back pain	[115]
	LL/BM/GM	-	To evaluate back pain and secondary lameness	[116]
	sEMG	To assess of m. longissimus electromyographic activity using sEMG—the high maximum activity of m. longissimus dorsi could contribute to the development of muscle pain at this site	-	[54,104,108,109]
IVDD	Algometry		To assess the intensity of back pain	[115]
	LL/BM/GM		To evaluate back pain and secondary lameness	[116]
	sEMG	To determine the presence of a peripheral neurogenic component to muscle atrophy, as muscle atrophy may be mistakenly presumed to be associated with disuse	**-**	[28]
DSSL/DISL	Algometry	-	To assess the intensity of back pain	[115]
	LL/BM/GM	-	To evaluate back pain and secondary lameness	[116]
	sEMG	-	Because of functional connection of SSL and ISL with m. longissimus dorsi suggests that sEMG studies on this muscle could be helpful for confirming the clinical importance of diagnostic imaging signs as well as for the follow-up of treatment and rehabilitation	[40,110]
LMS	Algometry	-	To assess the intensity of back pain	[115]
	LL/BM/GM	-	To evaluate back pain and secondary lameness	[116]
	sEMG	-	To observe sEMG amplitude between part of the body with a history of strain injury and uninjured contralateral part for evaluation the risk of reinjury	[118]
TIBP/RIBP	Algometry	-	To assess the intensity of back pain	[115]
	LL/BM/GM	To assess the shape of the back caused by pain	To evaluate back pain and secondary lameness	[6]
	sEMG	-	There is no research	**-**

BM, Biometric Mat; DISL, Desmopathy of the Intraspinous Ligament; DSSL, Desmopathy of the Supraspinous Ligament; FES, Functional Electrical Stimulation; GM, Geometric Morphometrics; IDSP, Impinging Dorsal Spinous Processes; IVDD, Intervertebral Discs Disease; LL, Lameness Locator; LMS, Longissimus Muscle Strain; OAAP, Osteoarthritis of Articular Process; RIBP, Rider Induced Back Pain; sEMS, surface Electromyography; TIBP, Tack Induced Back Pain; VS, Ventral Spondylosis.

## 5. Conclusions

The use of a conventional diagnostic protocol is necessary for the basic recognition of the main cases of equine back pain. However, an increasing number of measurement devices and methods, such as algometers, lameness locators, or undersaddle pressure mats, make it possible to objectify parts of the clinical examination. Moreover, the introduction of advanced diagnostic modalities, such as sEMG and FES, to clinical practice can be very useful in the functional assessment of the back and provide important clinical data that have rarely been considered so far. In equine medicine, the clinical applications of advanced diagnostic modalities seem promising for a more comprehensive evaluation of IDSP, IVDD, DSSL/DISL, and TIBP/RIBP.

## Data Availability

Not applicable.
